# A CMOS MEMS Humidity Sensor Enhanced by a Capacitive Coupling Structure

**DOI:** 10.3390/mi7050074

**Published:** 2016-04-26

**Authors:** Jian-Qiu Huang, Baoye Li, Wenhao Chen

**Affiliations:** Key Laboratory of MEMS of the Ministry of Education, Southeast University, Sipailou 2, Nanjing 210096, China; 220143654@seu.edu.cn (B.L.); 230149404@seu.edu.cn (W.C.)

**Keywords:** CMOS MEMS, capacitive humidity sensor, nanowires, coupling electrode

## Abstract

A capacitive coupling structure is developed to improve the performances of a capacitive complementary metal oxide semiconductor (CMOS) microelectromechanical system (MEMS) humidity sensor. The humidity sensor was fabricated by a post-CMOS process. Silver nanowires were dispersed onto the top of a conventional interdigitated capacitive structure to form a coupling electrode. Unlike a conventional structure, a thinner sensitive layer was employed to increase the coupling capacitance which dominated the sensitive capacitance of the humidity sensor. Not only static properties but also dynamic properties were found to be better with the aid of coupling capacitance. At 25 °C, the sensitive capacitance was 11.3 pF, the sensitivity of the sensor was measured to be 32.8 fF/%RH and the hysteresis was measured to be 1.0 %RH. Both a low temperature coefficient and a fast response (10 s)/recovery time (17 s) were obtained.

## 1. Introduction

The growing applications of humidity sensors in various areas such as agriculture, industry and household have promoted research in the design and fabrication technologies of humidity sensors [1,2,3,4]. There are many kinds of humidity sensors including electrolytic humidity sensors [5], resistive humidity sensors [6,7], optical humidity sensors [8,9], capacitive humidity sensors [10,11] and so on. Among them, capacitive humidity sensors are widely used because of advantages of low power consumption, good performances and long-term stability [4,12].

Capacitive humidity sensors are normally based on the humidity-dependent dielectric constant of a sensitive layer. Electrode geometry design has been studied in depth to optimize the performances of capacitive humidity sensors or other chemical sensors [13,14,15,16]. Generally, there are two kinds of capacitive humidity sensors: One is based on an interdigitated structure and the other is based on a parallel plate structure [17]. Interdigitated structures are widely used because of their simple fabrication processes [18,19,20,21,22]. In chemical sensors, interdigitated structures are usually applied because one side of these structures can be open to the ambient conditions. However, the sensitive capacitance and the sensitivity of an interdigitated capacitive structure are usually small [20,21,22]. Although finer geometry of the electrodes means higher sensitivity, small finger sizes are limited by the lithography process. As a result, much effort has been made to improve the structure of capacitive interdigitated sensors. In 2010, Lazarus *et al.* introduced a multi-stacked metal structure to enhance the sensitivity of an interdigitated humidity sensor [23]. However, the fabrication process was complex. In 2012, Kim *et al.* developed an interdigitated structure with increased height [24]. The sensitivity of the humidity sensor was enhanced but a thick sensitive layer was employed. It is well known that the sensitivity increases with the thickness of a sensitive layer while the hysteresis and the response/recovery time rise as well [13,25]. In our previous work, a poly-silicon heater was used to reduce both the hysteresis and the response/recovery time of a capacitive humidity sensor [26]. However, greater power consumption was required. To solve these problems, in this paper a capacitive coupling method was applied to improve the performances of an interdigitated capacitive humidity sensor. As a result, both static and dynamic properties were improved.

## 2. Structure and Operating Principle

**Figure 1:**
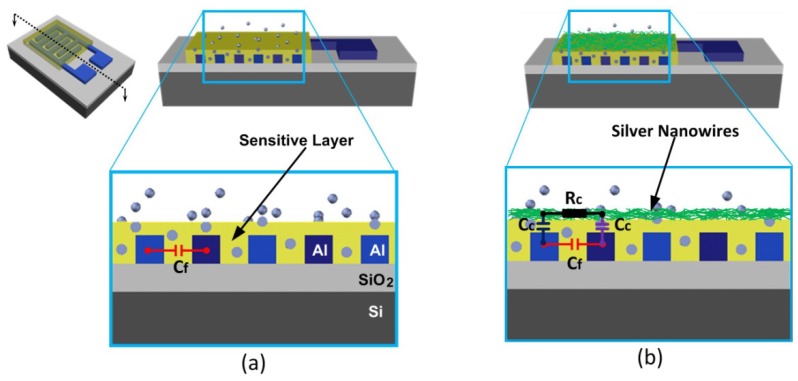
Sketch of an interdigitated capacitive humidity sensor: (**a**) conventional structure; (**b**) capacitive coupling structure.

The humidity sensor with a conventional structure and an improved structure are shown in Figure 1. A coupling electrode is employed in the improved structure to enhance the sensitive capacitance as well as the sensitivity. The coupling electrode is made of silver nanowire networks so that a grid structure is obtained to allow the penetration of water molecules. Although conductive nano-particles have been used in an interdigitated resistive structure to enhance the performance of a gas sensor [27], it is not practical for an interdigitated capacitive structure. In an interdigitated resistive structure, conductive nano-particles were employed as dispersed electrodes to reduce electrical losses in the sensitive material. However, in this paper the top electrode should be continuous to maximize the coupling capacitance between the two interdigitated electrodes. So that nanowires instead of nano-particles are used to form a continuous coupling electrode.

Unlike a conventional interdigitated structure, a thinner sensitive layer is preferred to increase both the coupling capacitance and the sensitivity of the sensor. Considering the limitation of the leakage current a 0.1–0.2 μm thick sensitive layer can be used which is 10–20 times thinner than that in a conventional interdigitated structure. However, in the improved structure, an additional nanowire layer is deposited on the top, so it is porous and the total thickness of the nanowire layer and the sensitive layer is still thinner. Therefore, the hysteresis and the response/recovery time are reduced. Actually, the capacitive coupling structure is a combination of an interdigitated structure and a parallel plate structure. In a conventional parallel plate structure, the connection between the top electrode and its corresponding pad should be carefully designed to avoid step coverage problems over different materials. As to the capacitive coupling structure the top electrode (nanowire networks in this paper) is not connected outside and it is just a floating electrode which is more robust than a conventional parallel plate structure.

**Figure 2:**
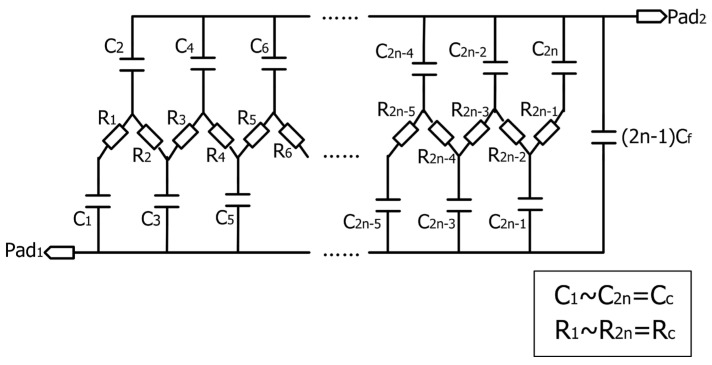
Equivalent circuit of an interdigitated capacitive humidity sensor with a nanowire coupling electrode.

Figure 2 shows the equivalent circuit of the capacitive coupling structure. Where *C*_f_ is the capacitance between adjacent fingers, *R*_c_ is the parasitic resistance of the coupling electrode between adjacent fingers and *C*_c_ is the coupling capacitance between each finger and the coupling electrode. The parasitic capacitance between the interdigitated electrode and the substrate are not included in this model because it can be eliminated by connecting the substrate to the ground [28]. With a thinner sensitive layer, *C*_c_ is usually much larger than *C*_f_ so that the sensitive capacitance is dominated by the coupling capacitance.

## 3. Fabrication Process

**Figure 3:**
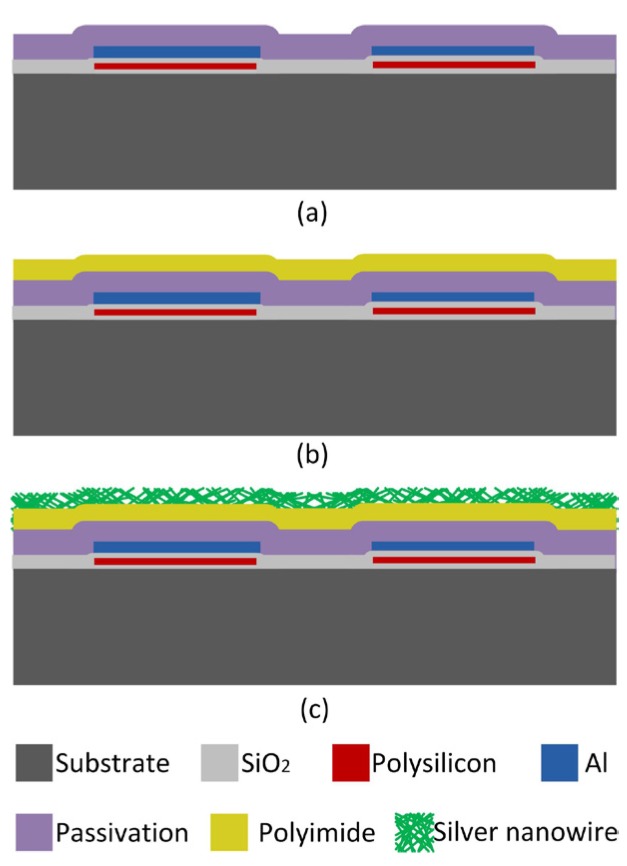
Fabrication process of the humidity sensor: (**a**) interdigitated structure fabricated by a complementary metal oxide semiconductor (CMOS) process; (**b**) structure after deposition of a polyimide film (**c**) structure after deposition of a silver nanowire electrode.

The humidity sensor with a capacitive coupling structure was fabricated by a post CMOS process. As shown in Figure 3a, standard 3-μm 1-poly-1-metal CMOS process was used to construct the interdigitated structure as described in our previous work [12]. First, a 0.045-μm thick silicon dioxide (SiO_2_) layer was thermal grown on a (100) p-type silicon (Si) wafer. Then a 0.45-μm thick poly-silicon (poly-Si) layer was deposited by a low pressure chemical vapor deposition (LPCVD) process. The poly-Si layer was patterned to form an integrated heater (the heater was not used in this paper). Next, an LPCVD insulating layer of SiO_2_ with a thickness of 0.55 μm was deposited and etched to form contact holes. Afterwards, a sputtered aluminum layer was used to fabricate a pair of interdigitated electrodes. The thickness of the electrodes was 1.2 μm. Finally, a 1-μm thick passivation layer was deposited and vias were opened in the pad regions.

Following the CMOS process, a two-step post process was applied. First, a 0.1-μm thick polyimide was spun onto the wafer to act as a humidity sensitive material (Figure 3b). A lithographic and dry etching process was used to remove the polyimide in the pad regions (which was not drawn in the figure). The polyimide was cured under nitrogen purge as follows: ramp to 150 in half an hour; hold at 150 °C for 1 h; ramp to 250 in half an hour; hold at 250 °C for 1 h; ramp to 350 °C in half an hour; hold at 250 °C for 1 h; cool to 20 °C during ~3 h. At last, silver nanowires were dispersed onto the polyimide with a SonoPlot GIX Microplotter instrument (SonoPlot, Inc., Middleton, WI, USA) to form the coupling electrode as shown in Figure 3c. The concentration of the silver nanowire ink used in this work was approximately 5 mg/mL and it was heated at 120 °C for 5 min after the dispersion process.

**Figure 4:**
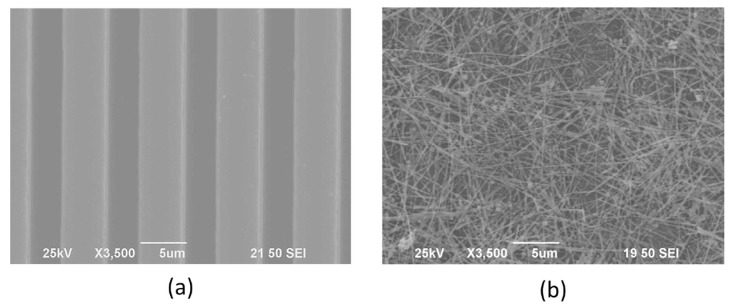
Scanning electron microscope (SEM) image of the fabricated structure before and after post-microelectromechanical system (MEMS) process: (**a**) interdigitated electrodes fabricated by a typical CMOS process; (**b**) silver nanowires deposited on the top of the structure.

The scanning electron microscope (SEM) image of the humidity sensor before and after the post process is shown in Figure 4. Figure 4a shows details of the interdigitated electrodes before the post process. In the structure, the width and length of each finger were 5 μm and 3200 μm, respectively. The spacing of adjacent fingers was 3 μm. The total area of the sensitive capacitor was 509 μm × 3200 μm. In Figure 4b, polyimide was spun on the interdigitated electrodes and silver nanowires were dispersed on the top of polyimide. The average diameter and length of the silver nanowires were 40 nm and 20 μm, respectively.

## 4. Results

### 4.1. Static Measurements

**Figure 5:**
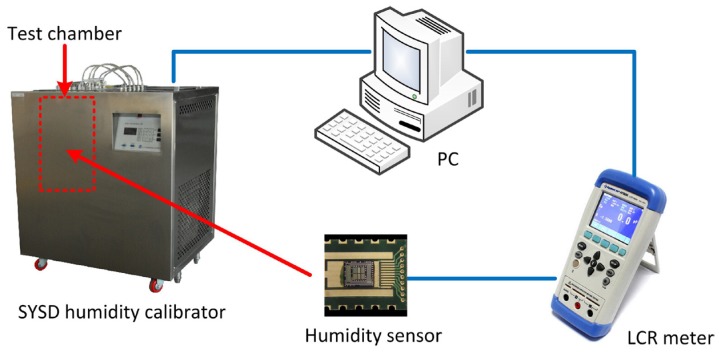
Test system of the humidity sensor for static measurements.

Static humidity measurements were performed using a humidity calibrator (Beijing Great Smart New Technology Co., Ltd., Beijing, China) as shown in Figure 5. The humidity calibrator consisted of a test camber, a thermostat bath and a dual-pressure humidity generator. The temperature inside the test chamber was stabilized by the thermostat bath and the humidity atmosphere was provided by the dual-pressure humidity generator. Both of them were controlled by a computer. An LCR meter (Changzhou Applent Technology Co., Ltd., Changzhou, China) was used to measure the sensitive capacitance of the humidity sensor. The test frequency was 100 kHz and the test results were recorded by the computer.

**Figure 6:**
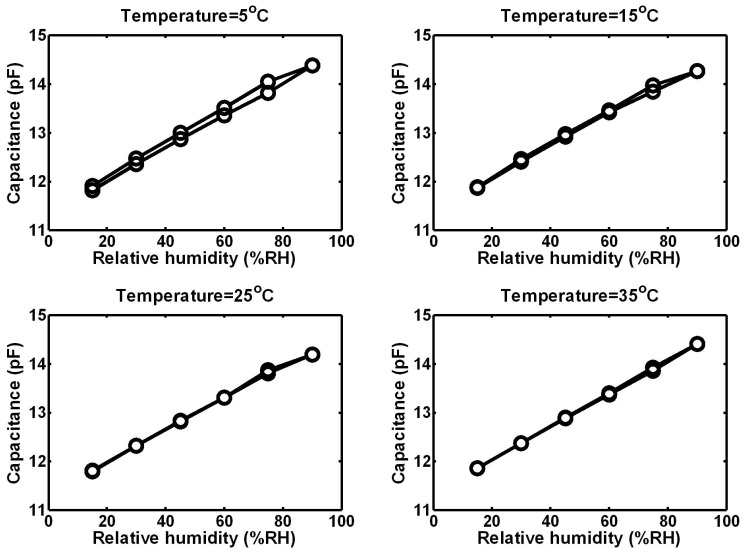
Output of the humidity sensor as a function of relative humidity at different temperatures.

The humidity sensor with a capacitive coupling structure was calibrated from 15 to 90 %RH at 5, 15, 25 and 35 °C, respectively. Both an adsorption process and a desorption process were carried out. The humidity response curves of the sensor are shown in Figure 6. The sensor exhibited good sensitivity and linearity. The temperature coefficient of the sensor was not significant over a range of temperatures from 5 to 35 °C. The hysteresis of the sensor reduced with the temperature because of the promotion of diffusion. It is clear that water molecules can escape from the sensitive material more easily with higher temperature. The maximum hysteresis was 3.5 %RH occurred at 75 %RH and 5 °C. Table 1 shows details of static properties of the humidity sensor.

### 4.2. Dynamic Measurements

**Figure 7:**
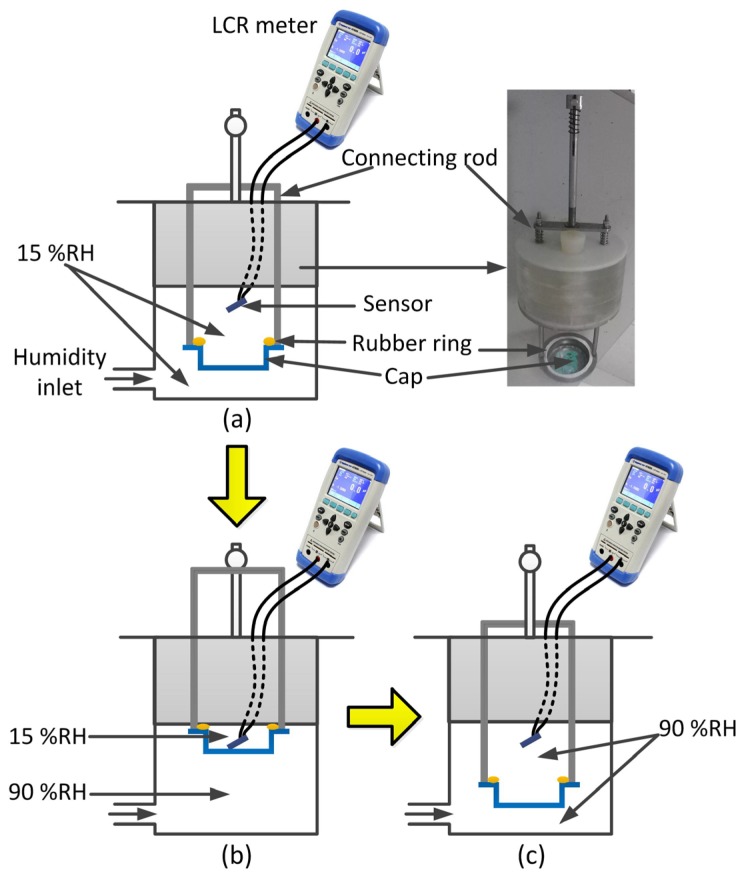
Test system of the humidity sensor for dynamic measurements. (**a**) The first step of the measurement; (**b**) The second step of the measurement; (**c**) The third step of the measurement.

As description in Figure 7, an experiment set up was constructed to evaluate the dynamic properties of the humidity sensor. Both a response process and a recovery process were performed at 25 °C. Take the measurement of response time, for example. First, the relative humidity in the chamber was set to be 15 %RH. A humidity sensor was fixed in the test chamber and allowed to stabilize (Figure 7a). Then the cap inside the test chamber was pulled upwards by the connecting rod as shown in Figure 7b. The humidity sensor was sealed in a 15 %RH atmosphere. Next, the humidity outside the cap increased to 90 %RH and kept for 5 min. Finally, the cap was pushed downwards quickly to achieve an abrupt increase of relative humidity (15–90 %RH). The sensitive capacitance was measured by an LCR meter and recorded by a computer.

**Figure 8:**
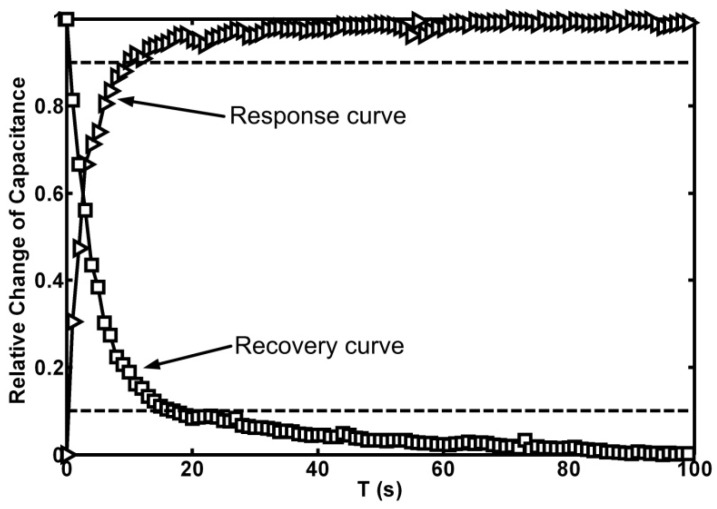
Dynamic test results of the sensor at 25 °C.

Both a response curve and a recovery curve of the humidity sensor are illustrated in Figure 8. The response time is defined as the time for the sensitive capacitance to rise to 90% of its final steady-state value and the recovery time is defined as the time for the sensitive capacitance to fall to 10% of its initial vale. As described in Figure 8, the response time of the improved structure was 10 s and the corresponding recovery time was 17 s.

## 5. Discussion

**Figure 9:**
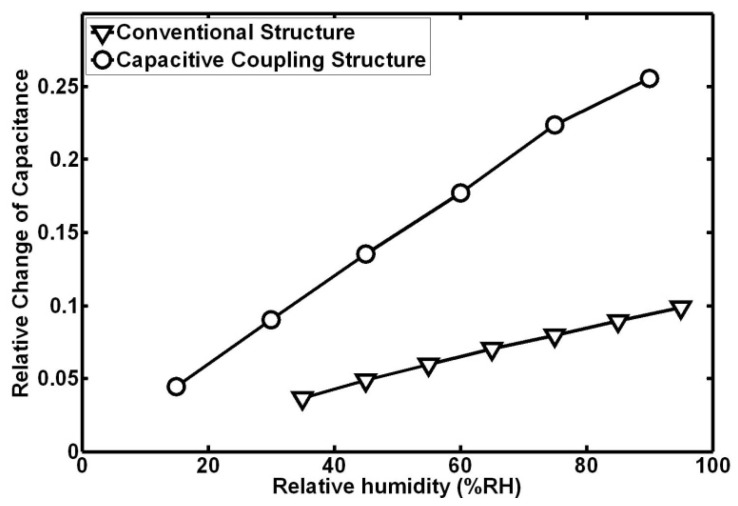
Relationship between relative change of capacitance and relative humidity at 25 °C.

As described in our previous work, a conventional interdigitated capacitive humidity sensor suffered from low sensitivity (1.46 fF/%RH at 25 °C) and significant hysteresis. An on-chip heater was introduced to reduce the hysteresis of the sensor (decreased to 1 %RH at 25 °C) but it had little effect on the sensitivity [24]. To evaluate the sensitivity of different structures, the output should be characterized by a relative change of capacitance Δ*C*/*C*_dry_, where *C*_dry_ is the sensitive capacitance in a dry air atmosphere. In a conventional interdigitated structure, *C*_dry_ is about 1.4 pF and in a capacitive coupling structure, *C*_dry_ is about 11.3 pF. Figure 9 shows the relationships between relative change of capacitance and relative humidity of both structures at 25 °C. In percentage terms, the sensitivity increases from 0.1 %/%RH to 0.29 %/%RH with the aid of a capacitive coupling effect. In other words, the sensitivity of the capacitive coupling structure is 2.9 times higher than that of a conventional structure. Additionally, the response time of a conventional structure is 38 s (without self-heating) and 25 s (with self-heating). The response time of a capacitive coupling structure is 10 s, which is about 3.8 times faster than that of a conventional structure and 2.5 times faster than that of a self-heating structure.

The capacitive coupling structure is compared with both a multi-stacked metal structure and an increased height structure as shown in Table 2. For all of the structures the sensitivity is enhanced and the capacitive coupling structure is more efficient than the other two structures. Both the capacitive coupling structure and the multi-stacked metal structure exhibit better linearity than the increased height structure. The hysteresis of the increased height structure is larger than that of the capacitive coupling structure because the former one uses a thicker sensitive layer. The recovery time of the multi-stacked metal structure is slower than that of the capacitive coupling structure. Both the capacitive coupling structure and the multi-stacked metal structure are fabricated by a CMOS MEMS process but the latter one is much more complex.

## 6. Conclusions

A capacitive coupling technique was used to improve the performances of an interdigitated capacitive structure in a CMOS MEMS humidity sensor. The sensitive capacitance of the sensor was enhanced by the coupling capacitance. A thinner sensitive layer was used to increase the coupling capacitance and the sensitivity. The humidity sensor was fabricated by a post-CMOS process. Both static and dynamic measurements were carried out. According to the measurements, the sensitivity of the sensor was 32.8 fF/%RH (0.29 %/%RH in percentage terms) and the hysteresis of the sensor was 1 %RH at 25 °C. The temperature coefficient of the sensor was not significant over a range of temperature from 5 to 35 °C. The response time of the improved structure was 10 s and the recovery time was 17 s at 25 °C. With the aid of the capacitive coupling structure, the sensitivity increases 2.9 times and the response time reduces 3.8 times as compared to that of a conventional structure. In the future work, the humidity sensor will be further improved by optimizing the thickness of films, the concentration of the nanowire ink and the sizes of nanowires.

## Figures and Tables

**Table 1:** Static properties of the humidity sensor with a capacitive coupling structure.

Property	5 °C	15 °C	25 °C	35 °C
Sensitivity (fF/%RH)	33.9	32.7	32.8	33.9
Linearity	1.5%	3.6%	2.7%	0.4%
Hysteresis (%RH)	3.5	1.9	1.0	1.0

**Table 2:** Comparison of different optimized structures.

Structure	Improvement of Sensitivity	Linearity	Hysteresis	Recovery Time
Capacitive coupling structure	2.9 times	good	1.0 %RH	17 s
Multi-stacked metal structure [23]	2 times	good	Not reported	>20 s
Increased height structure [24]	2.3 times	poor	2.87 %RH	Not reported

## References

[1] Farhani H., Wagiran R., Hamidon M.N. (2014). Humidity sensors principle, mechanism and fabrication technologies: A comprehensive review. Sensors.

[2] Stanislav A.K., Neil T.G., Chengbo M., Kaiming Z. (2014). Toward a new generation of photonic humidity sensors. Sensors.

[3] Chia-Yen L., Gwo-Bin L. (2005). Humidity sensors: A review. Sens. Lett..

[4] Chen Z., Lu C. (2005). Humidity sensors: A review of materials and mechanisms. Sens. Lett..

[5] Dunmore F. (1938). An electric hygrometer and its application to radio meteorography. J. Res. Natl. Bur. Stand..

[6] Doroftei C., Popa P.D., Iacomi F. (2012). Study of the Influence of Nickel Ions Substitutes in Barium Stannates Used as Humidity Resistive Sensors. Sens. Actuators A Phys..

[7] Lim D.-I., Cha J.-R., Gong M.-S. (2013). Preparation of Flexible Resistive Micro-Humidity Sensors and Their Humidity-Sensing Properties. Sens. Actuators B Chem..

[8] Lin Q., Li Y., Yang M. (2012). Polyaniline Nanofiber Humidity Sensor Prepared by Electrospinning. Sens. Actuators B Chem..

[9] Imran Z., Batool S.S., Jamil H., Usman M., Israr-Qadir M., Shah S.H., Jamil-Rana S., Rafiq M.A., Hasan M.M., Willander M. (2013). Excellent Humidity Sensing Properties of Cadmium Titanate Nanofibers. Ceram. Int..

[10] Li J., Lin X., Li J., Liu Y., Tang M. (2012). Capacitive Humidity Sensor with a Coplanar Electrode Structure Based on Anodised Porous Alumina Film. Micro Nano Lett..

[11] Dean R.N., Rane A.K., Baginski M.E., Richard J., Hartzog Z., Elton D.J. (2012). A Capacitive Fringing Field Sensor Design for Moisture Measurement Based on Printed Circuit Board Technology. IEEE Trans. Instrum. Meas..

[12] Fenner R., Zdankiewicz E. (2001). Micromachined water vapor sensors: A review of sensing technologies. IEEE Sens. J..

[13] Rittersma Z.M. (2002). Recent Achievements in Miniaturised Humidity Sensors—A Review of Transduction Techniques. Sens. Actuators A Phys..

[14] Ribeiro L.E.B., de Alcântara G.P., Andrade C.M.G., Fruett F. (2015). Analysis of the Planar Electrode Morphology Applied to Zeolite Based Chemical Sensors. Sens. Transducers.

[15] Rivadeneyra A., Fernández-Salmerón J., Banqueri J., López-Villanueva J.A., Capitan-Vallvey L.F., Palma A.J. (2014). A novel electrode structure compared with interdigitated electrodes as capacitive sensor. Sens. Actuators B Chem. B.

[16] Kim J.H., Hong S.M., Moon B.M., Kim K. (2010). High-Performance Capacitive Humidity Sensor with Novel Electrode and Polyimide Layer Based on MEMS Technology. Microsys. Technol..

[17] A Comparison of Relative Humidity Sensing Technologies. http://pasternack.ucdavis.edu/files/6213/7271/8210/hyd151_read13.pdf.

[18] Dai C.L. (2007). A capacitive humidity sensor integrated with micro heater and ring oscillator circuit fabricated by CMOS-MEMS technique. Sens. Actuators B Chem..

[19] Nizhnik O., Higuchi K., Maenaka K. (2012). Self-calibrated humidity sensor in CMOS without post-processing. Sensors.

[20] Gu L., Huang Q.A., Qin M. (2004). A novel capacitive-type humidity sensor using CMOS fabrication technology. Sens. Actuators B Chem..

[21] Rivadeneyra A., Fernández-Salmerón J., Agud M., López-Villanueva J.A., Capitan-Vallvey L.F., Palma A.J. (2014). Design and characterization of a low thermal drift capacitive humidity sensor by inkjet-printing. Sens. Actuators B Chem..

[22] Oprea A., Bâarsan N., Weimar U., Bauersfeld M.L., Ebling D., Wollenstein J. (2008). Capacitive humidity sensors on flexible RFID labels. Sens. Actuators B Chem..

[23] Lazarus N., Bedair S.S., Lo C.C., Fedder G.K. (2010). CMOS-MEMS capacitive humidity sensor. J. Microelectromech. Syst..

[24] Kim J.H., Moon B.M., Hong S.M. (2012). Capacitive Humidity Sensors Based on a Newly Designed Interdigitated Electrode Structure. Microsyst. Technol..

[25] Igreja R., Dias C.J. (2006). Dielectric response of interdigital chemocapacitors: The role of the sensitive layer thickness. Sens. Actuators B Chem..

[26] Zhao C.L., Qin M., Huang Q.A. (2011). A fully packaged CMOS interdigital capacitive humidity sensor with polysilicon heaters. IEEE Sens..

[27] Tricoli A., Pratsinis S.E. (2010). Dispersed Nanoelectrode Devices. Nat. Nanotechnol..

[28] Schrode D.K. (2006). Semiconductor Material and Device Characterization.

